# Impact of COVID-19 pandemic on foot care services in Ontario, Canada

**DOI:** 10.1186/s13047-022-00555-2

**Published:** 2022-06-24

**Authors:** Suzanne H. Lu, Ann-Marie McLaren, Ellie Pinsker

**Affiliations:** 1 Wound Care Team, Surgery & Critical Care Program, Unity Health Toronto - St. Michael’s Hospital, 30 Bond Street, Toronto, ON M5B 1W8 Canada; 2 Li Ka Shing Knowledge Institute, Unity Health Toronto, Toronto, ON Canada; 3 Division of Orthopaedic Surgery, University of Toronto, Unity Health Toronto – St. Michael’s Hospital, Toronto, ON Canada

**Keywords:** COVID-19, Foot care, Chiropodists, Podiatrists, Foot health, Telehealth/virtual care, Mental health, Pandemic preparedness

## Abstract

**Background:**

The COVID-19 pandemic has directly affected the delivery of health care services in Canada, including foot care. The goal of this descriptive study was to understand the impact of the early COVID-19 pandemic (March 2020 to April 2021) on chiropodists’ and podiatrists’ clinical practices and foot care service delivery in Ontario, Canada.

**Methods:**

A web-survey was completed by participating chiropodists and podiatrists registered with the College of Chiropodists of Ontario. The survey consisted of 31 multiple choice and open-ended items on clinical practice characteristics, foot care service delivery changes, perceived barriers during the pandemic, and its impact on clinicians. Descriptive statistics were used to characterize the sample and examine clinicians’ responses, and qualitative content analysis was used to explore opened-ended items.

**Results:**

Of the 773 eligible clinicians, 279 participated for a response rate of 36.1%. Most respondents reported a decline in patient volume, an increase in urgent foot health problems, a financial impact on their clinical practices, an emotional impact, and substantial changes to how they provided foot care services, such as incorporating telehealth/virtual care into patient care. Factors that impact clinicians’ perception of future pandemic preparedness are identified.

**Conclusion:**

This study describes foot care service delivery in Ontario, Canada during the COVID-19 pandemic. The COVID-19 pandemic saw an increase in urgent foot health problems, decline in patient volume, and impacted clinicians’ mental health and emotional well-being. Future studies should examine patients’ experiences of foot care service delivery and maintaining their foot health during the pandemic, and further examination of factors that impact clinicians’ perception of pandemic preparedness.

## Background

**Fig. 1:**
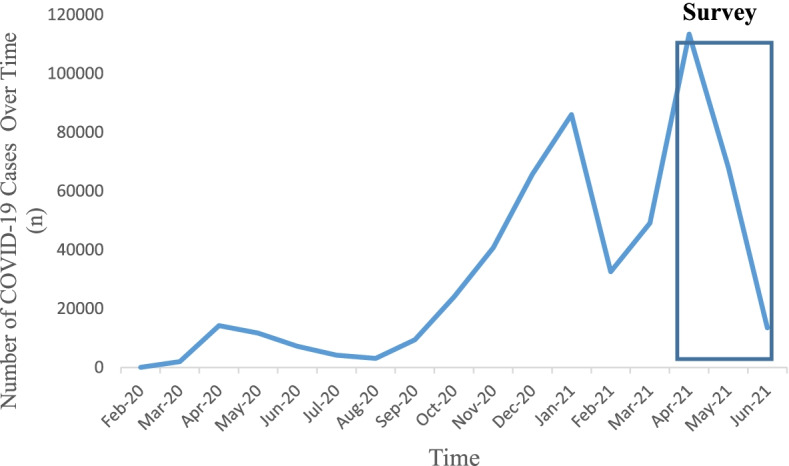
Number of COVID-19 Cases in Ontario from March 2020 to June 2021 (As reported by Ontario Ministry of Health, All Ontario: Case numbers and spread, https://covid-19.ontario.ca/data/case-numbers-and-spread, Accessed 8th July, 2021.)

On March 11, 2020, the World Health Organization (WHO) announced that the rapidly spreading novel coronavirus (COVID-19) had reached pandemic levels [1]. In Ontario, the most populous Canadian province, over half a million individuals contracted COVID-19 and there were approximately 9,100 deaths attributed to the virus as of June 30, 2021 [2]. During these 16 months, the province experienced three waves of COVID-19 that altered the delivery of health care (Fig. 1. Number of COVID-19 Cases in Ontario from March 2020 to June 2021) [2].

On March 19, 2020, the Chief Medical Officer of Health of Ontario (CMOHO) issued a directive for all health care providers requiring all non-essential and elective services be ceased or reduced to minimal levels, which impacted the provision of foot care services in the province [3, 4]. At this time, there were close to 800 chiropodists and podiatrists collectively providing foot care services to over 14 million Ontario residents [5, 6]. Practicing chiropodists and podiatrists in the province are regulated by the College of Chiropodists of Ontario (COCOO). For the purpose of this study, chiropodists and podiatrists will be referred to as foot care providers (FCPs).

Following the CMOHO directive, COCOO defined essential care as “urgent foot health problems”, which included sudden onset of pain, increasing signs of infection, and diabetic foot changes during this time of service interruption [7]. As non-essential foot care services restarted in June 2020, the COCOO recommended a careful and gradual restart to services and introduced new clinical practice guidelines for FCPs. These guidelines detailed safety measures to limit and prevent the spread of COVID-19 among patients, clinicians, and staff in clinical practices [3, 8]. The purpose of the current study was to understand how the COVID-19 pandemic impacted foot care service delivery, patient foot health, and the mental health and well-being of FCPs practicing in Ontario, Canada.

## Methods

An exploratory cross-sectional study using internet-based survey methods was conducted to provide an accurate and detailed description of clinician services provided during the COVID-19 pandemic. Internet-based survey delivery is cost-effective and enables data to be retrieved in a timely and efficient manner [9]. All active members of the COCOO (94% chiropodists, 6% podiatrists) providing foot care services in the province of Ontario in April 2021 were eligible for inclusion (*n* = 773) (with the exception of the study’s authors) [5]. Clinician members were contacted by COCOO via email with a description of the study and a web link to the online survey, which was administered through an online survey platform (SurveyMonkey®). Initial survey questions focused on characterizing clinicians’ practices, while later questions examined their experiences providing foot care services during the COVID-19 pandemic (March 2020 through April 2021) (Fig. 1), including FCPs perceptions of patient volume, barriers to care, their mental health and emotional well-being. The survey consisted of 31 items, including 5 open-ended items and 26 multiple-choice items with the option to provide additional comments. Surveys were available to potential participants for an 8-week period beginning in April 2021. All participants were emailed by COCOO with a reminder to complete the survey after one month. The survey was anonymous with no identifying information collected. The study was approved by the Unity Health Toronto-St. Michael’s Hospital Research Ethics Board (REB#20–251). Accessing the web link and completion of the survey served as informed consent.

Descriptive statistics (frequencies, means and percentages) were used to report and characterize responses. Statistical analyses were performed with SPSS Version 28.0 (Chicago, IL). Open-ended responses and additional written comments to multiple choice were analyzed using an inductive approach to qualitative content analysis as described by Elo et al. and Hsieh et al. [10, 11].

## Results 

### Participants

There were 773 registered clinicians eligible for inclusion. Two hundred and seventy-nine completed the survey, resulting in a 36.1% response rate. Basic information on participating clinicians and their clinical practices can be found in Table 1. The majority of participants (207, 74.5%) worked in a private clinic setting. Most had been in practice for 21 to 30 years (77, 27.8%) and were located in “District 1—Toronto (100, 36.0%) or “District 5—Central East” (78, 28.1%). These jurisdictions contain municipalities with the largest populations in the province. Participants were closely split between those who worked in a single practice or institution (156, 56.5%), versus those with a secondary practice (120, 43.5%).

**Table 1:** Clinical Practice Information (*n* = 279)

Characteristic	Number of Clinicians n (%)
**Years in Practice**	
0–5 years	54 (19.5)
6–10 years	50 (18.1)
11–20 years	41 (14.8)
21–30 years	77 (27.8)
> 30 years	55 (19.9)
**Electoral District**	
District 1—Toronto	100 (36.0)
District 2—South West	26 (9.4)
District 3—Central West	30 (10.8)
District 4—East	27 (9.7)
District 5—Central East	78 (28.1)
District 6—North	17 (6.1)
**Primary Care Setting**	
Private clinic	207 (74.5)
Community health center or family health team	51 (18.3)
Hospital	12 (4.3)
Other^a^	8 (2.9)
Practitioners with a second practice, n (% yes)	120 (43.5)

^a^Other: nursing homes, long-term care, patients’ homes, educational/school clinics

### Impact on clinicians’ practices

Survey participants described the impact of the COVID-19 pandemic on foot care services in a variety of ways. In particular, they experienced a decrease in patient volume (189, 68.0%), an increase in the cost of running their practices (163, 58.6%), reduced access to podiatric equipment and supplies (150, 54.0%), and increased wait times for patients (147, 52.9%) (Table 2). The biggest barriers, according to respondents, were scheduling of patients to allow for physical distancing (110, 39.6%), additional time to provide care (92, 33.1%), increased operating costs (92, 33.1%), and what clinicians perceived as patient anxiety or reluctance to return to care (172, 61.9%) (Table 2). During the first wave of the pandemic (March to August 2020), the provincial government declared a shortage of personal protective equipment (PPE) from medical distribution channels and COCOO notified members of a known shortage of accessible PPE. However, by April 2021, 41.0% (*n* = 114) reported access to PPE as a barrier, with access to disposable surgical or procedure masks (55, 19.8%), disposable gloves (53, 19.1%), and N95 masks (49, 17.6%) representing the PPEs most difficult to obtain (Table 2).

**Table 2:** Clinicians’ Experiences with Foot Care Delivery During COVID-19 Pandemic

	**Number of Clinicians** **n (%)**
**Experienced Impact of Pandemic on Provision of Care**	
Decreased patient volume	189 (68.0)
Increased operating costs	163 (58.6)
Reduced access to podiatric equipment and supplies	150 (54.0)
Increased wait times for patients	147 (52.9)
Lacked PPE	114 (41.0)
Increased wait times for specialist referrals	101 (36.3)
Increased need for patient triage	101 (36.3)
Reduced access to diagnostic tests	80 (28.8)
Reduced staffing	80 (28.8)
Increased fees for patients	67 (24.1)
**Perceived Barriers to Providing Care**	
Patients’ anxiety/reluctance to return to care	172 (61.9)
Scheduling	110 (39.6)
Operating costs	92 (33.1)
Time to provide care	92 (33.1)
Patient screening	58 (20.9)
Staffing	52 (18.7)
Clinicians’ anxiety to provide care	33 (11.9)
Infection control practices	32 (11.5)
Limited PPE	16 (5.8)
**Experienced Difficulty Accessing Personal Protective Equipment**	
Disposable surgical or procedure masks	55 (19.8)
Disposable gloves	53 (19.1)
N95 masks	49 (17.6)
Gel hand sanitizer	36 (12.9)
Disposable gowns	33 (11.9)
Facial protective shields	31 (11.2)
**Instituted Precautionary Measures**	
Prescreened patients	215 (77.3)
Increased sanitizing practices in clinic	210 (75.5)
Provided PPE for clinicians/staff	204 (73.4)
Rescheduled patients	190 (68.3)
Increased wait times between patients	180 (64.7)
Installed physical structures (e.g. glass barrier, air filtration)	172 (61.9)
Updated infection control policies/procedures	170 (61.2)
Requested patient wait outside clinic/treatment area (not in waiting room)	166 (59.7)
Provided PPE for patients	152 (54.7)
Introduced contact-tracing protocol	134 (48.2)
Patients completed COVID-19 consent and/or waiver forms	114 (41.0)
Took patients’ temperatures prior to clinic entry	104 (37.4)

*PPE* Personal Protective Equipment

Participants’ responses indicated a consistent decline in patient volume as compared to pre-pandemic patient capacity levels (Table 2). Specifically, 36.1% of respondents reported seeing over 76 patients per week prior to the pandemic, while only 18.8% have reported seeing high patient volume during the pandemic (up to April 2021). The greatest changes were observed for clinicians seeing less than 25 patients per week and over 100 patients per week pre-pandemic. Conversely, only 30.5% of respondents saw fewer than 50 patients per week pre-pandemic, which increased to 61.7% during the pandemic. By April 2021, 36.2% of respondents reported no change in the number of patients compared to their pre-pandemic patient volume (Table 3). In terms of the financial impact of COVID-19 on clinicians’ practices, the majority of respondents reported a moderate to great impact (46.5% versus 38.7%), while only 14.8% of clinicians (*n* = 32) reported minimal to no impact.

**Table 3:** Number of Patients seen Weekly by Clinicians (*n* = 261)

	**Pre-Pandemic** **n (%)**	**April 2021** **n (%)**
< 25 patients	32 (1.8)	94 (36.0)
26–50 patients	78 (28.7)	67 (25.7)
51–75 patients	64 (23.5)	51 (19.5)
76 to 100 patients	54 (19.9)	35 (13.4)
> 100 patients	44 (16.2)	14 (5.4)

### Changes to foot care service delivery

Following the cessation of care which lasted from March 19, 2020 to May 26, 2020, the CMOHO encouraged all health care providers “to implement a system for virtual and/or telephone consultations when and where possible” [3]. Initial consultations conducted by virtual or telephone consultation would determine whether an in-person appointment was necessary. This would also support physical distancing efforts and limit contact of individuals who may potentially have had COVID-19 with others in healthcare settings. One hundred and twenty participants (55.3%) reported incorporating telehealth and/or virtual care into their practices. Specifically, 80 (36.9%) used telephone only, 2 (0.9%) used video (i.e. virtual care) only, and 38 (17.5%) used both. Ninety-seven (44.7%) did not change their practice to include telehealth or virtual care. Participants commented on a reluctance to implement virtual care as they felt the need to provide direct hands-on interventions in order to provide effective, appropriate and safe foot care. As the pandemic wore on, many moved to some form of virtual care while some participants reported that virtual care was not possible due to barriers faced by patients not having a phone, equipment, or sufficient internet access.

The CMOH also encouraged health care providers to introduce precautionary measures and modifying the delivery of services [4]. Study participants reported a variety of modifications which they introduced in their clinical practices (Table 2). There was broad uptake of these safety precautions, which notably included: pre-screening of patients (i.e. travel history, any contact with a COVID-19 positive individual, presence of any COVID-19 symptoms) (215, 77.3%), increasing sanitizing practices in the clinic environment (210, 75.5%), providing PPE to clinicians and other healthcare setting staff (204, 73.4%), rescheduling patients who reported being COVID-19 positive or reported potential COVID-19 symptoms (190, 68.3%), increasing wait times between patients (180, 64.7)%, and installation of physical structures (e.g. sinks, ventilation, glass or plexiglass, etc.) (172, 61.9%).

Despite the increase in costs associated with many of the modifications to service delivery and the reduction in patient capacity, only 67 (24.1%) of respondents reported increasing fees to patients (Table 2). In the open-ended comments, numerous respondents communicated their intention to continue with the precautionary measures and augmented infection control practices in their clinical practices post-pandemic.

### Impact on patient foot health

Due to the halt of all non-emergent surgeries and non-urgent health care services (from March through May 2020), participants found it necessary to prioritize in-person care for urgent foot health problems after restarting non-essential services in June 2020. In April 2021, 197 (83.8%) respondents reported that they had been contacted by patients or seen patients regarding an urgent foot health problem since the start of the pandemic. Furthermore, 131 (55.5%) respondents reported seeing an increase in the number of patients with foot health problems between November 2020 and April 2021. The type and number of urgent foot health problems varied greatly by respondent. One hundred and sixty-nine clinicians saw patients for pain-limiting mobility (60.6%), 183 for infection (65.6%), 176 for wounds (63.1%), 150 for diabetic foot changes (53.8%), and 164 for self-inflicted injuries sustained while trying to provide their own foot-care (58.8%) (Table 4). Respondents also reported treating foot health issues, such as ingrown toenails, tendonitis, foreign body, trauma, and gangrene. Fifty-six clinicians (23.8%) described at least one patient with a non-traumatic or diabetes-related lower extremity amputation during the pandemic.

**Table 4:** Urgent Foot Health Problems (*n* = 279)

**Type of Foot Health Problem**	**Clinicians with ≥ 1 Patient Experiencing a Foot Health Problem** **n (%)**	**Number of Patients** **(Mean ± SD, range)**
Infection	183 (65.6)	25.0 ± 46.8 (0–400)
Wound	176 (63.1)	10.9 ± 14.0 (0–80)
Pain limiting mobility	169 (60.6)	38.7 ± 68.5 (0–500)
Self-care causing injury	164 (58.8)	22.0 ± 39.7 (0–250)
Diabetic foot change	150 (53.8)	17.8 ± 31.4 (0–250)
Foot health problem requiring medical care from a primary care physician and/or nurse practitioner	145 (52.0)	12.5 ± 32.9 (0–300)
Foot health problem requiring emergency department visit and/or hospitalization	113 (40.5)	5.4 ± 15.3 (0–150)

*SD* Standard Deviation

### Mental health and emotional well-being

Survey respondents expressed that the COVID-19 pandemic had affected their mental health and emotional well-being in a variety of ways: stress (162, 58.3%), fatigue (139, 50.0%), anxiety (128, 46.0%), burnout (90, 43.4%), fear (54, 19.4%), and depression (53, 19.1%). Only 15 individuals (5.4%) reported no impact. Sixty-two respondents (28.6%) sought support or accessed other resources to improve their mental health and physical wellness in the time encompassed by this survey (up to April 2021).

In April 2021, 149 respondents (68.3%) reported feeling prepared for subsequent COVID-19 waves, 186 (86.1%) felt prepared for future pandemics, and 181 (83.4%) thought that vaccines would return their practices to their pre-pandemic state. Respondents also indicated that vaccines, stable supplies of PPE, public health crisis guidelines, and clear provincial and federal responses are important factors that contribute to their feeling prepared for future pandemics.

## Discussion

This is the first study to report on the delivery of foot care services by chiropodists and podiatrists in Ontario and examines their experiences during a pandemic. In an effort to limit the transmission of COVID-19 in the community, FCPs pivoted their care delivery by incorporating measures mandated by the provincial government and COCOO [3, 8]. Our study illustrates the significant impact of the COVID-19 pandemic on the delivery of foot care services in the province, the surge of urgent foot health problems, and the emotional, mental, and financial impact on FCPs during the study period.

FCPs in Ontario have a fiduciary responsibility to follow mandates by their governing body. During the pandemic, FCPs closely adhered to these mandates including: pre-screening patients for symptoms of COVID-19, scheduling patients to allow for physical distancing, increasing sanitization of the clinical environment, and providing PPE for themselves, staff, and patients [8]. Survey respondents reported that scheduling, additional time to provide care, and increased operating costs were the greatest barriers to delivering foot care during the first three waves of the pandemic, along with patients’ trepidation to return to care. However, respondents expressed in the open-ended items that the practice changes contributed to their feeling optimistic and confident about their capability to provide foot care services during the current pandemic. Many indicated they will maintain these care delivery changes post-pandemic.

In our study, FCPs reported seeing a substantial decline in patient volume as a result of recommended changes in the provision of care. Scheduling was the most reported barrier due to screening requirements and physical distancing guidelines in support of safe delivery of services [3]. Clinicians also posited the decline to reluctance of their patients to receive in-person care. Patients had communicated their anxiety and fear to return for in-person foot care to their foot care providers. This reported drop in patient volume is consistent with literature published during this pandemic. Researchers in Slovenia reported a decline of 58% in clinic visits [12], while Shin et al. reported the number of patient encounters was reduced by 50% in Manchester, UK, and nearly 70% in Los Angeles, USA [13].

To offset the lack of in-person care, over half of FCPs incorporated telephone consultations and/or virtual care into their practices by the third wave of the pandemic. Many FCPs reported the challenge of providing care in this manner due to the important visual component inherent in foot care and patients lacking necessary technology or equipment to facilitate virtual care. Similarly promoted use of virtual care was described by Rogers et al. when they were unable to provide in-person care for their patients with foot ulcers in the USA [14]. In contrast, the majority of Australian podiatrists did not incorporate telehealth into their practice, continuing instead with high-risk in-person care [15]. The authors explained that there was limited evidence to support the use of virtual care in the context of general foot care and that funding had not been provided to public podiatry clinics to support its introduction [15].

Given the decline in patient volume and poor uptake of virtual care in clinical practices, FCPs anticipated a substantial increase in the number of diabetic foot problems as the pandemic continued. Diabetes mellitus is a chronic disease that is associated with poor foot outcomes, including peripheral neuropathy, structural foot deformity, foot ulceration, and lower leg amputation [16, 17]. Patients with this condition are at a higher risk for these common and morbid disease complications, necessitating routine monitoring and foot care [18]. Accordingly, over half of FCPs reported seeing patients with diabetic foot changes. FCPs also described an increase in the number of in-person visits for urgent foot health problems. They indicated that their patients sought foot-related care from their primary care physicians, and patients visited the Emergency Department for urgent foot-related problems. Almost a quarter of clinicians disclosed that at least one patient had undergone a non-traumatic or diabetes-related lower extremity amputation during the pandemic. These urgent foot health problems represent a potentially preventable burden on Ontario’s public health care system.

With regard to the financial impact of the COVID-19 pandemic on their clinical practices, the majority of FCPs described greater operating costs, including necessary PPE, sanitization supplies, and installation of physical structures. At the same time, the majority of FCPs were treating fewer patients. Despite these practice changes, only 24% of FCPs increased their treatment costs for patients. This may be because FCPs did not want to deter patients from returning to foot care or create additional barriers for patients at a time when many were experiencing a financial strain due to the pandemic.

Along with the described financial impact of the COVID-19 pandemic on the clinical practices of FCPs, providing patient care during a pandemic has also been shown to emotionally impact health care professionals, including the mental health and well-being of FCPs. In a systematic review of the mental impact of the COVID-19 pandemic on healthcare workers, Muller et al. reported that healthcare workers experienced anxiety, depression, and sleep problems during the pandemic [19]. Similarly, Evanoff et al. found that working in a clinical environment was associated with higher anxiety and decreased well-being [20]. In the current study, FCPs described similar experiences, including stress, fatigue, anxiety, and burnout. Muller et al. also found that healthcare workers were less likely to seek professional help [19]. More than half of FCPs in this study did not reach out for support or access resources to improve their mental health and physical wellness.

Despite the emotional and financial burden experienced by FCPs, they also communicated their adaptability and preparedness to face ongoing practice changes. This may have been influenced by the vaccination efforts underway, as well as the diminishing number of active COVID-19 cases in the province at the time of this survey. Furthermore, the implementation of physical structures, technological platforms, and enhanced infection control practices contributed to respondents’ confidence in delivering foot care services during another pandemic in the future. Finally, numerous respondents discussed their impressions of what factors are essential for future pandemic preparedness, including the guarantee of an adequate stockpile of PPE, coordinated communication from all government levels, and the creation of a practice crisis preparedness checklist or manual. These factors would minimize the disruption to delivery of foot care services during future global crises.

Given that there is no previously published literature describing the clinical practice of FCPs in Ontario, Canada, this study’s findings cannot be compared to pre-pandemic practice norms. The survey included both podiatrists and chiropodists, however, information was not collected to allow comparison by FCP designation. The study also employed a web-based survey methodology, which is known to have a lower response rate and may be affected by responder bias [9, 21, 22]. Nevertheless, the authors chose this methodology for its ease, speed, reach, affordability, and convenience. The response rate was consistent with other studies utilizing web-based surveys [15, 23].

Future research could examine hospital resource utilization data (e.g. hospital admissions and lower extremity amputations related to foot complications) and foot care procedure data from primary care practitioners to understand the overall impact of COVID-19 on the healthcare system. In addition, this study has highlighted areas for future research for more thorough understanding of clinician’s emotional well-being using validated questionnaires. As well, a qualitative research study would help gain insight into the patients’ perspective on the provision of foot care services during the pandemic. This would be important to further understand the impact on patients and explore the factors raised by FCPs that would better prepare health care providers for future pandemics.

## Conclusion

This study describes the delivery of foot care services by chiropodists and podiatrists in Ontario, Canada during the COVID-19 pandemic. Foot care providers pivoted their clinical practice in accordance with governing guidelines as they dealt with increasing numbers of urgent foot health problems while patient volume declined. There was an impact on the mental, emotional and financial well-being of foot care providers as they adapted to continuing changes. Pandemic preparedness is necessary to provide foot care services and to navigate through future global epidemics.

## Data Availability

Request for further data and materials may be submitted to Suzanne Lu (Suzanne.Lu@unityhealth.to). Aggregate or summarized data used during the current study may be shared based on reasonable request.

## Abbreviations

COVID-19: Novel coronavirus
WHO: World Health Organization
CMOHO: Chief Medical Officer of Health of Ontario
COCOO: College of Chiropodists of Ontario
FCPs: Foot care providers
PPE: Personal protective equipment

## References

[1] World Health Organization (2020). WHO director-general’s opening remarks at the media briefing on COVID-19.

[2] Ontario Ministry of Health (2021). All Ontario: case numbers and spread.

[3] Ontario Ministry of Health (2020). COVID-19 operational requirements: health sector restart version 2..

[4] College of Chiropodists of Ontario (2020). COVID-19 communications.

[5] College of Chiropodists of Ontario (2021). Member communications.

[6] Statistics Canada (2021). Population estimates quarterly.

[7] College of Chiropodists of Ontario (2021). Questions and answers for members of the public regarding care during the COVID-19 pandemic.

[8] College of Chiropodists of Ontario (2020). COVID-19 pandemic clinical practice directive version 3.0.

[9] Fan W, Yan Z (2010). Factors affecting response rates of the web survey: a systematic review. Comput Hum Behav.

[10] Elo S, Kyngäs H (2008). The qualitative content analysis process. J Adv Nurs.

[11] Hsieh HF, Shannon SE (2005). Three approaches to qualitative content analysis. Qual Health Res.

[12] Urbančič-Rovan V (2021). Diabetic foot care before and during the COVID-19 epidemic: what really matters?. Diabetes Care.

[13] Shin L, Bowling FL, Armstrong DG, Boulton AJM (2020). Saving the diabetic foot during the COVID-19 pandemic: a tale of two cities. Diabetes Care.

[14] Rogers LC, Lavery LA, Joseph WS, Armstrong DG. All feet on deck – the role of podiatry during the COVID-19 pandemic: preventing hospitalizations in an overburdened healthcare system, reducing amputation and death in people with diabetes. J Am Podiatr Med Assoc. 2020:0000. 10.7547/20.051. 10.7547/20-051 32208983

[15] Williams CM, Couch A, Haines T, Menz HB (2021). Experiences of Australian podiatrists working through the 2020 coronavirus (COVID-19) pandemic: an online survey. J Foot Ankle Res.

[16] Jaly I, Iyengar K, Bahl S, Hughes T, Vaishya R (2020). Redefining diabetic foot disease management service during COVID-19 pandemic. Diabetes Metab Syndr.

[17] Rodrigues BT, Vangaveti VN, Urkude R, Biros E, Malabu UH (2022). Prevalence and risk factors of lower limb amputations in patients with diabetic foot ulcers: a systematic review and meta-analysis. Diabetes Metab Syndr.

[18] Bus SA, Lavery LA, Monteiro-Soares M, Rasmussen A, Raspovic A, Sacco ICN, van Netten JJ, International Working Group on the Diabetic Foot (2020). Guidelines on the prevention of foot ulcers in persons with diabetes (IWGDF 2019 update). Diabetes Metab Res Rev..

[19] Muller AE, Hafstad EV, Himmels JPW, Smedslund G, Flottorp S, Stensland SØ, Stroobants S, Van de Velde S, Vist GE (2020). The mental health impact of the covid-19 pandemic on healthcare workers, and interventions to help them: a rapid systematic review. Psychiatry Res.

[20] Evanoff BA, Strickland JR, Dale AM, Hayibor L, Page E, Duncan JG, Kannampallil T, Gray DL. Work-related and personal factors associated with mental well-being during the COVID-19 response: survey of health care and other workers. J Med Internet Res. 2020;22(8):e21366. 10.2196/21366. Erratum in: J Med Internet Res. 2021;23(4):e29069. 10.2196/21366 PMC7470175 32763891

[21] Kidd JC, Colley S, Dennis S (2021). Surveying allied health professionals within a public health service: what works best, paper or online?. Eval Health Prof.

[22] Manfreda KL, Berzelak J, Vehovar V, Bosnjak M, Haas I (2008). Web surveys versus other survey modes: a meta-analysis comparing response rates. Int J Mark Res.

[23] Normahani P, Mustafa C, Standfield NJ, Duguid C, Fox M, Jaffer U (2018). Management of peripheral arterial disease in diabetes: a national survey of podiatry practice in the United Kingdom. J Foot Ankle Res.

